# Synthesis of Zinc Oxide Nanoparticles From Aqueous Extract of Avicennia marina Mangrove Leaves and Their Antibacterial Activities Against Oral Pathogens

**DOI:** 10.7759/cureus.47627

**Published:** 2023-10-25

**Authors:** Shabnam Tamanna I, Sivaperumal Pitchiah, Vasugi Suresh, Pasiyappazham Ramasamy

**Affiliations:** 1 Physiology, Saveetha Dental College and Hospitals, Saveetha Institute of Medical and Technical Sciences, Saveetha University, Chennai, IND; 2 Prosthodontics, Saveetha Dental College and Hospitals, Saveetha Institute of Medical and Technical Sciences, Saveetha University, Chennai, IND

**Keywords:** innovative technique, novel agents, uv spectroscopy, oral pathogens, avicennia marina, zn nanoparticles, biosynthesis

## Abstract

Introduction

The field of nanotechnology is currently being extensively researched. Nanoparticles (NPs) are used in many fields, such as engineering and medicine, owing to their nanoscale dimensions. Zinc (Zn) appears to be the most desirable metal NP, as it is being applied in various drug delivery systems and other fields. The green synthesis of the NPs used in this study makes it affordable and nonpolluting. *Avicennia marina *leaves possess antimicrobial properties and a high secondary metabolite content. This study aimed to synthesize ZnO NPs from the aqueous extracts of *A. marina *mangrove leaves and assess their antibacterial activities against oral pathogens.

Methodology

The leaves of *A. marina* were dried to obtain a preprocessed powder, and from that, an aqueous extract was prepared. ZnO NPs were then synthesized by adding the aqueous extract to 100 mL of ZnS solution and mixing it in an orbital shaker. They were observed both visually and by ultraviolet (UV) spectrophotometry to confirm their synthesis. The antibacterial properties of these ZnO NPs were assayed using the disc diffusion method on three different oral bacterial strains (*Streptococcus mutans*, *Staphylococcus aureus, *and *Klebsiella *sp*.*).

Results

For the synthesis process, it was seen that zinc oxide (ZnO) NPs exhibited a deepening in coloration. Additionally, the UV spectrum analysis revealed a notable absorbance value of 1.2 at a wavelength of 320 nm. The antibacterial efficacy against *S. mutans*, *S. aureus*, and *Klebsiella* sp. was assessed by measuring the zone of inhibition in diameter. At a dosage of 100 µg/mL of ZnO NPs, the inhibition zones were found to be 7.5 ± 0.2, 9.5 ± 0.5, and 9.5 ± 1.2 mm for *S. mutans*, *S. aureus*, and *Klebsiella* sp., respectively. Similarly, at a concentration of 75 µg/mL, the inhibition zones were measured to be 7 ± 0.25, 9 ± 1, and 7.5 ± 0.5 mm for the respective bacterial strains.

Conclusions

This study synthesizes ZnO NPs using *A. marina* leaf aqueous extract in a sustainable and eco-friendly manner. The ZnO NPs' antibacterial activities against oral infections indicate their use in dental products. These NPs have promising potential for nanomedicine and oral health studies due to their antibacterial properties and ecologically sustainable manufacturing.

## Introduction

Nanotechnology is an upcoming field in science and engineering, where occurrences at the nanometer scale are utilized in the design, application, manufacturing, and characterization of various appliances and systems. Nanoparticles (NPs) act as links between bulk materials and atomic or molecular structures [1]. Their extraordinary and interesting properties result from their small size, enormous surface area with free dangling bonds, and more reactivity than their mass cousins. [2]. NPs are usually synthesized using physical and chemical methods under high pressure, energy, harmful chemicals, and high temperatures. Plants and plant extract-based green syntheses are cost-effective and eco-friendly. Researchers are becoming more interested in biological processes owing to the development of effective green synthesis using natural reducing, capping, and stabilizing agents without the use of harmful, expensive chemicals, and all with low energy usage [3-5]. Because it is affordable and highly effective and has an easy synthesis approach at an industrial scale, the green synthesis of NPs using plant extracts has grown remarkably in recent years [6-9]. There is no demand for the use of high concentrations of surfactants or polymers for green synthesis.

Nano-ZnS has peculiar physical and chemical characteristics that set it apart from bulk ZnS, including a higher surface-to-volume ratio, quantum size effect, surface and volume effect, and macroscopic quantum tunneling effect. They also absorb more light, exhibit greater chemical activity and thermal resistance, are catalytic, and have a lower melting point. Among all other metal NPs, ZnO NPs are the most feasible because of their use in drug delivery systems, biosensors, cosmetics, biomedicine, and agriculture. Recently, these NPs have been used in wastewater management, textiles, and medicine [10]. Mangroves are a type of medicinal plant, and extracts from various parts of the plant are extensively utilized worldwide [11]. *Avicennia marina* is a species of mangrove tree that belongs to the Avicenniaceae plant family and is commonly referred to as the white or gray mangrove [12]. This plant can be found in the Persian Gulf-based Iranian mangrove forests of Qeshm. Rheumatism, smallpox, abscesses, and ulcers have all traditionally been treated with *A. marina*'saerial parts of *A. marina*. Other infectious disorders have been treated using this plant in traditional Persian medicine. In addition to its traditional uses, crude extracts of this plant have been shown to have certain pharmacological effects, including antibacterial properties and the ability to induce cancer cell apoptosis [13]. Marine plants grow in different environments than terrestrial plants; therefore, it is suspected that mangrove plants have different characteristics that would account for the production of various types of bioactive compounds [14,15]. *A. marina* leaves have been shown to possess antibacterial, antiplasmodial, and antiviral activities. They are also found to have a high content of secondary metabolites, such as polyphenols, flavonoids, alkaloids, and tannins [16]. Accordingly, the present investigation aimed to synthesize ZnO NPs from *A. marina* leaves and assess their antibacterial properties against oral pathogens. This study will aid in finding a greener and more effective way to fight the known hazards caused by microbial populations.

## Materials and methods

Preparation of *A. marina* leaf powder

**Figure 1:**
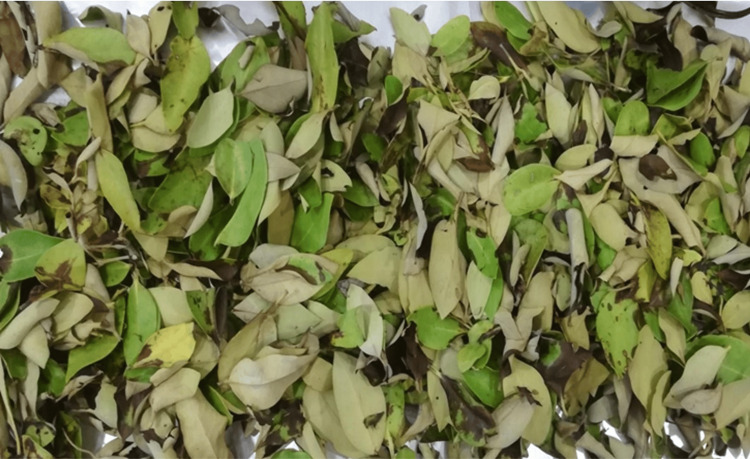
Avicennia marina mangrove leaf sample. The figure is taken from our laboratory while drying the leaf sample.

The mangrove leaves *A. marina*, were collected from the Tuticorin coast located at latitude 8°44'57.6"N and longitude 78°11'07.1"E in Tamil Nadu, India. The leaves were then preprocessed by cleaning and washing with distilled water. This preprocessed sample was then dried in a hot-air oven at temperatures below 60 °C. Subsequently, the dried sample was crushed into a coarse powder using a mortar and pestle (Figure 1). 

Preparation of aqueous extract of *A. marina* leaf powder

**Figure 2:**
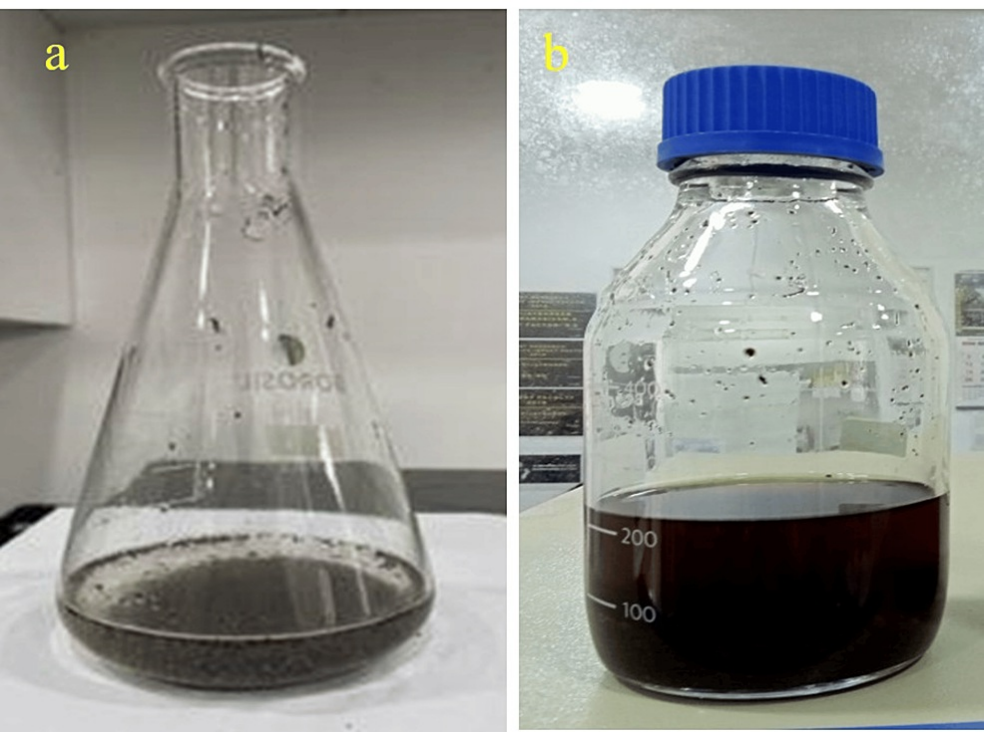
(a) Aqueous extract of Avicennia marina mangrove leaf: (b) crude extract.

The powdered sample (50 g) was mixed with 100 mL of distilled water in a conical flask and kept in an orbital shaker with 180 rpm for 24 h. This extract was then filtered through a muslin cloth and concentrated using a rotary evaporator to obtain the crude extract (Figure 2).

Synthesis of ZnO NPs

An aqueous solution of ZnS (10 mM) was prepared using double-distilled water. ZnS solution of 100 mL was then placed in a conical flask, and about 5 to 10 mL of aqueous extract, which was previously prepared, was added dropwise while continuously stirring in an orbital shaker. The biosynthesized solution was observed visually and examined further using a ultraviolet (UV) spectrophotometer with a wavelength in the range of 200-800 nm [17]. The biosynthesized samples were then centrifuged at 12,000 rpm. The pellets were separated and placed in a hot-air oven at 65 ℃ for 24 h.

Antibacterial activity of ZnO NPs

The antibacterial activity of ZnO NPs was assessed using the disc diffusion method. Whatman filter paper discs (5 mm) were impregnated with various concentrations of NPs. Inoculate nutritional agar plates with *Klebsiella* sp., *Staphylococcus aureus*, and *Streptococcus mutans*, three common oral bacterial pathogens. Use a sterile cork borer to create wells in the agar plates. To completely scatter the produced ZnO NPs, a measured quantity is dissolved in deionized water and then sonicated. ZnO NPs at 75 and 100 µg/mL concentrations should be added to the agar wells. The plates should be incubated at 37 ℃ for 24 h. To evaluate ZnO NPs' efficacy as an antibacterial agent against oral infections, we can measure the diameters of their respective zones of inhibition. Three independent samples' means and standard deviations are reported. As a positive control, we utilized tetracycline (10 g/disc).

## Results

Visual observation

**Figure 3:**
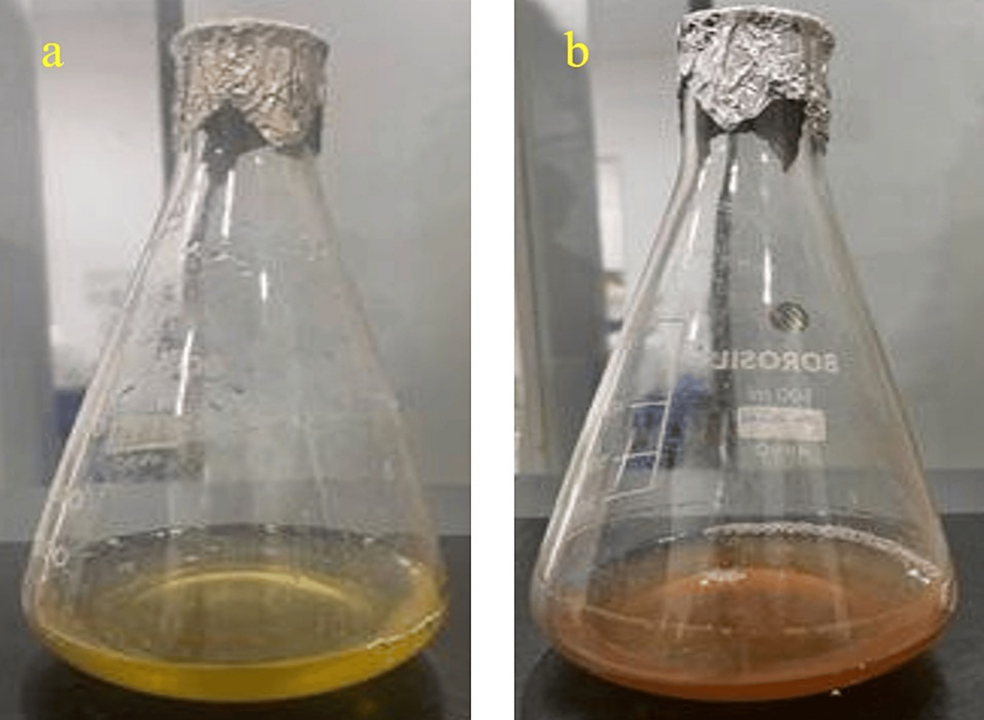
(a) Initial stage of ZnO nanoparticle synthesis and (b) color change after 24 h. ZnO, zinc oxide

As shown in Figure 3, the solution containing ZnS and the aqueous extract was observed for 24 h, and it was observed that the color became darker, indicating the synthesis of ZnO NPs.

UV visible spectroscopy of ZnO NPs

**Figure 4:**
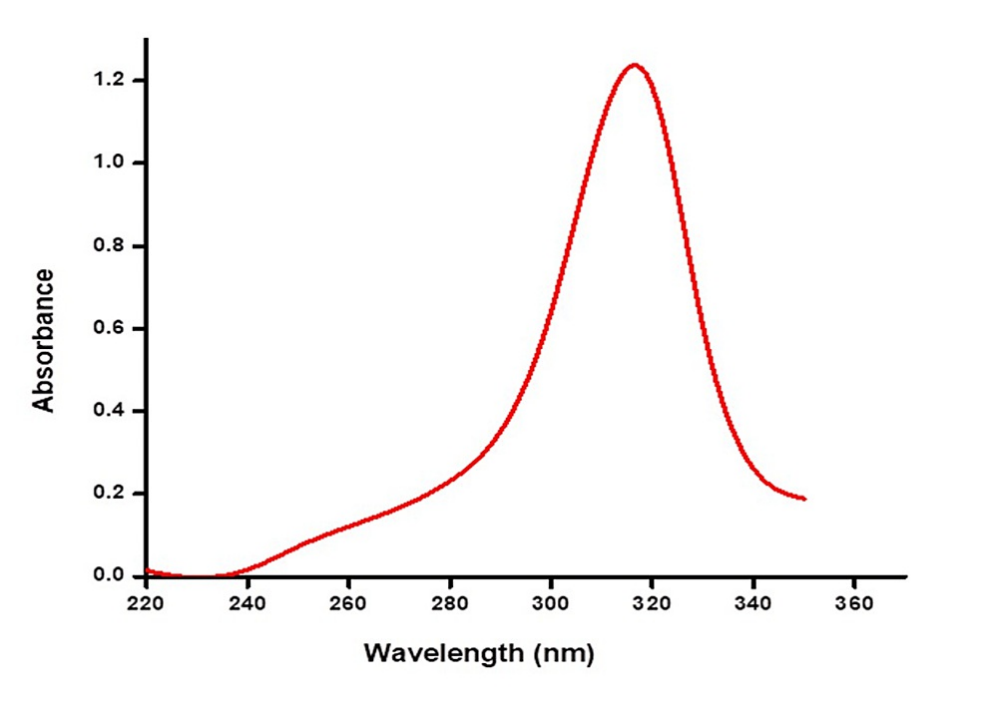
UV visible spectroscopy of ZnO nanoparticles. ZnO, zinc oxide; UV, ultraviolet

The UV spectrum graph in Figure 4 shows that the ZnO NPs synthesized from* A. marina *mangrove leaves exhibited a maximum absorbance of 1.2 at a wavelength of 320 nm.

Antibacterial activity

**Figure 5:**
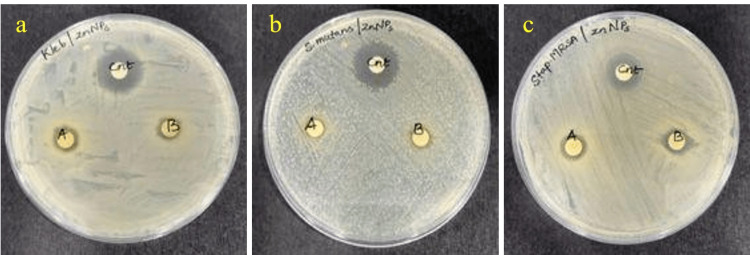
Antibacterial activity of ZnO nanoparticles on (a) Klebsiella sp., (b) Staphylococcus aureus, and (c) Streptococcus mutans. ZnO, zinc oxide

The antibacterial activity of green-synthesized ZnO NPs against three different oral pathogens, *Klebsiella* sp., *S. aureus*, and *S. mutans*, was assessed by measuring their zone of inhibition around the discs from the back of the plate (Figure 5). The NPs exhibited excellent antibacterial activity at two concentrations, with inhibition zones for *S. mutans*, *S. aureus*, and *Klebsiella* sp. measuring 7.5 ± 0.2, 9.5 ± 0.5, and 9.5 ± 1.2 mm at a ZnO NP concentration of 100 µg/mL. Furthermore, at a concentration of 75 µg/mL, the inhibition zones were 7 ± 0.25, 9 ± 1, and 7.5 ± 0.5 mm for the respective bacteria (Table 1).

**Table 1:** Inhibition zone by ZnO nanoparticles on three different oral pathogens: Klebsiella sp., Staphylococcus aureus, and Streptococcus mutans. MRSA, Methicillin-resistant *Staphylococcus aureus*; ZnO, zinc oxide

Nanoparticle concentration (µg/mL)	*Klebsiella *sp. (mm)	*Staphylococcus aureus* (MRSA) (mm)	*Streptococcus mutans* (mm)
100	9.5 ± 1.2	9.5 ± 0.5	7.5 ± 0.2
75	7.5 ± 0.5	9 ± 1	7 ± 0.25

## Discussion

ZnS NPs were green-synthesized in this study, instead of using physical, chemical, and hazardous methods. According to reports, green NPs have increased antibacterial activity compared to chemically generated NPs because most plants used in such studies often have antimicrobial capabilities [18]. The darkening of the color observed visually for 24 h indicated ZnO NP synthesis. Similar results were observed in another study, where the appearance of a dark brown color denoted the synthesis of Ag (silver) NPs synthesis from *A. marina* [19]. The bioactive components present in the extract appear to be responsible for reducing Ag metal ions to Ag NPs, which is why the color appears to change [20]. Indicating the different phytometabolites present in the leaves and flowers of *Calotropis gigantea*, one study reported the visual observation to display a difference in color change between Ag NP solutions synthesized from both parts, where the leaf extract-synthesized NP turned blackish-brown and that of flower extract turned yellow-brown [21]. In another study in which ZnO NPs were synthesized using *Plectranthus odoratissimum* leaf extract, the color changed from light red to cream, indicating the biosynthesis of ZnO [22]. The UV spectrum showed a maximum absorbance of 320 nm. A previous study showed that zinc NPs biosynthesized from *Deverra tortuosa* aqueous extract exhibited the highest absorbance at 374 nm [23]. In another study, ZnO NPs were synthesized from *Atalantia monophylla* leaf extract with a maximum absorbance peak at 352 nm [24]. Ag ions from *Mentha piperita* showed the highest absorbance at 420 nm in a previously conducted study [25]. Another study showed that nickel oxide NPs synthesized using *A. marina* exhibited a peak in the UV absorption spectrum at 297 nm [26]. In another study, the UV spectrum peak was observed at 295 nm, which proved that the synthesis of platinum NPs using *Atriplex hamilus* was successfully achieved [27]. A previous study reported a maximum absorbance peak at 370 nm for ZnO NPs synthesized using *P. odoratissimum* aqueous leaf extract (ALE) [22].

Observation of the inhibition zone of *A. marina*-synthesized ZnO NPs reveals that the most significant antibacterial activity, with inhibition zones of 9.5 ± 1.2 and 9 ± 1 mm, was observed against *Klebsiella* sp. at 100 µg/mL and *S. aureus* at 75 µg/mL, respectively. Overall, *S. aureus* was inhibited the best at both 100 and 75 µg/mL, with an inhibition zone of 9.5 ± 0.5 and 9 ± 1 mm. Previous studies reported that the biosynthesized ZnO NPs from *Pseudomonas aeruginosa* exhibited high efficacy against *S. aureus*, with an inhibition zone of 12.33 ± 0.9 mm [28], whereas in *A. marina*-mediated ZnO NP, the inhibition zone was 9.5 ± 0.5 and 9 ± 1 mm for two different concentrations of *S. aureus*. In another study, biosynthesized Ag NPs from the leaf extract of *A. marina* exhibited an inhibition zone of only 10.87 ± 1.33 mm against *S. aureus* [29]. In contrast, ZnO NPs synthesized from the same plant demonstrated inhibition zones for three pathogens: *S. aureus*, *S. mutans*, and *Klebsiella* sp. (9.5 ± 0.5; 9 ± 1 mm), (7.5 ± 0.2; 7 ± 0.25 mm), and (7.5 ± 0.2; 7 ± 0.25 mm), respectively. Another study showed that Ag/Fe_2_O_3_ NPs at 5 g/mL had a good antibacterial effect on *S. aureus*, with an inhibition zone of 22.3 ± 0.57 mm [30]. Similarly, in another study where Cu NPs were synthesized from *Kigelia africana* fruit, the antibacterial assay showed a striking inhibition zone of 8.0±2.83 mm on *S. aureus *[31]. Using the Mueller-Hinton agar method, one study showed that Pt NPs prepared using *Atriplex hamilus* leaves had an inhibition zone of 17 mm for *Klebsiella pneumonia* [27]. A previous study showed that ZnO NPs synthesized using *P. odoratissimum* leaf extract had a maximum inhibition zone of 28 ± 0.35 mm for *S. aureus* at a ZnO NP concentration of 10 µg/mL [22].

Limitations

This study, albeit with excellent results, is still in its primary stage and must be taken further by conducting more advanced assays to intricately characterize and understand the mechanism of action of these NPs. This study was limited to only three oral pathogens, with a small sample size. There is a need to test these biosynthesized ZnO NPs in various types of microorganisms that inhabit the oral mucosa. The research should go into detail about the characterization techniques utilized to confirm the synthesis of ZnO NPs, such as Fourier Transform Infrared Spectroscopy (FTIR), scanning electron microscopy (SEM), and X-ray diffraction (XRD). It is difficult to establish the quality, size, shape, and purity of NPs without extensive investigation. In vivo testing, which is required to understand how these NPs act in a real organism, is not included in the study. It would be beneficial to investigate potential adverse effects, tissue reactions, and actual efficacy within the oral cavity.

## Conclusions

In conclusion, ZnO NPs darkened in coloration. A recorded absorbance value of 1.2 was observed at a wavelength of 320 nm in the UV spectrum. The zone of inhibition diameter was measured to determine antibacterial activity against *S. mutans*, *S. aureus*, and *Klebsiella *sp. At 100 µg/mL ZnO NPs, the inhibition zones for *S. mutans*, *S. aureus*, and *Klebsiella* sp. were 7.5 ± 0.2, 9.5 ± 0.5, and 9.5 ± 1.2 mm, respectively. Similarly, at 75 µg/mL, the bacterial strains had inhibitory zones of 7 ± 0.25, 9 ± 1, and 7.5 ± 0.5 mm, respectively. The antibacterial properties of ZnO NPs have been demonstrated through in vitro investigations, suggesting their potential application in in vivo studies. Due to their notable efficacy, ZnO NPs have the potential to be employed in the pharmaceutical industry for drug delivery. ZnO NPs can also be used in the marine industry to produce natural products. Owing to their unique characteristics, metal NPs have been applied not only in the field of medicine but also in catalysis, textile engineering, nanobiotechnology, bioengineering sciences, optics, electronics, and water treatment.
